# Right ventricular systolic dysfunction and remodelling in Nigerians with peripartum cardiomyopathy: a longitudinal study

**DOI:** 10.1186/s12872-016-0204-8

**Published:** 2016-01-29

**Authors:** Kamilu Musa Karaye, Krister Lindmark, Michael Henein

**Affiliations:** Department of Medicine, Bayero University and Aminu Kano Teaching Hospital, 3 New Hospital Road, Kano, Nigeria; Department of Public Health and Clinical Medicine, Umea University, SE-901 87 Umea, Sweden; Department of Cardiology, Umea Heart Centre, SE-901 87 Umea, Sweden

**Keywords:** Peripartum cardiomyopathy, Right ventricular dysfunction, RV remodelling

## Abstract

**Background:**

The literature on right ventricular systolic dysfunction (RVSD) in peripartum cardiomyopathy (PPCM) patients is scanty, and it appears that RV reverse remodelling in PPCM has not been previously described. This study thus aimed to assess RVSD and remodelling in a cohort of PPCM patients in Kano, Nigeria.

**Methods:**

A longitudinal study carried out in 3 referral hospitals in Kano, Nigeria. Consecutive PPCM patients who had satisfied the inclusion criteria were recruited and followed up for 12 months. RVSD was defined as the presence of either tricuspid annular plane systolic excursion (TAPSE) <16 mm or peak systolic wave (S’) tissue Doppler velocity of RV free wall <10 cm/s. For the purpose of this study, recovery of RV systolic function was defined as an improvement of reduced TAPSE to ≥16 mm or S’ to ≥10 cm/s, without falling to reduced levels again, during follow-up.

**Results:**

A total of 45 patients were recruited over 6 months with a mean age of 26.6 ± 7.0 years. RV systolic function recovery occurred in a total of 8 patients (8/45; 17.8 %), of whom 6 (75.0 %) recovered in 6 months after diagnosis. The prevalence of RVSD fell from 71.1 % at baseline to 36.4 % at 6 months (*p* = 0.007) and 18.8 % at 1 year (*p* = 0.0008 vs baseline; *p* = 0.41 vs 6 month). Patients with RVSD had higher serum creatinine, and TAPSE accounted for 19.2 % (*p* = 0.008) of the variability of serum creatinine at 6 months. Although 83.3 % of the deceased had RVSD, it didn’t predict mortality in the regression models (*p* > 0.05).

**Conclusion:**

RVSD and reverse remodelling were common in Nigerians with PPCM, in whom the first 6 months after diagnosis seem to be critical for RV recovery and survival.

## Background

Peripartum cardiomyopathy (PPCM) is an important cause of heart failure (HF) in many parts of the world including Northern Nigeria, and is associated with significant morbidity and mortality [1, 2]. We previously described right ventricular (RV) systolic dysfunction in PPCM patients using tricuspid annular plane systolic excursion (TAPSE), and reported RV systolic dysfunction (RVSD) in 54.6 % of the patients [3]. It is believed that left ventricular (LV) function recovers in 23–41 % of PPCM patients over time, but the literature on RVSD in PPCM is still scanty and to the best of our knowledge, RV reverse remodelling in PPCM has not been previously described [2]. We hypothesised that many PPCM patients would also experience RV reverse remodelling over time. The present study thus aimed to assess RVSD and reverse remodelling over 1 year in a cohort of PPCM patients in Kano, Nigeria.

## Methods

This is a longitudinal study carried out in Murtala Mohammed Specialist Hospital (MMSH), Aminu Kano Teaching Hospital (AKTH) and a private cardiology clinic in Kano, Nigeria.

### Clinical evaluation

The study conformed to the ethical guidelines of the Declaration of Helsinki, on the principles for medical research involving human subjects [4]. The research protocol was approved by the Research Ethics Committees of AKTH and Kano State Hospitals Management Board before the study started. Inclusion criteria were: (i) new diagnosis of PPCM before commencement of medical treatment; (ii) onset of HF symptoms between last few months of pregnancy and first 5 months postpartum, (iii) at least 18 years of age; (iv) contact telephone number, except patients who gave reassurance that they were willing to attend the follow-up, and (v) giving written informed consent. We excluded PPCM patients who were on HF treatment, as well as those who presented more than 5 months since delivery. PPCM was defined according to the recommendations of the HF Association of the European Society of Cardiology Working Group on PPCM, and LV systolic dysfunction was defined as LV ejection fraction (LVEF) <45 % [2].

At the study sites, physicians and obstetricians were invited to refer all patients with suspected PPCM to the principal investigator (PI) for further evaluation. Patients were then interviewed, clinically evaluated and recruited consecutively. For each subject, a 12-lead electrocardiogram (ECG) at rest and trans-thoracic echocardiogram were carried out by the PI at the study centres according to standard recommendations [5]. The echocardiographic examination was carried out using Sonoscape S8 Doppler Ultrasound System (Shenzhen, China, 2010). Plasma hemoglobin and serum urea, electrolytes and creatinine were measured at the laboratories of AKTH according to standard protocols.

The PI re-evaluated the patients at 6 and 12 months follow-up, using the same protocol as at recruitment including ECG and echocardiographic examinations, but blood tests were not repeated.

#### Cardiac function assessment

Echocardiography was performed according to standard recommendations [5, 6]. RV basal diameter (RVb), Right atrial longitudinal dimension (RAL) and RA end-systolic area (RAA) were measured in each patient. Tricuspid annular plane systolic excursion (TAPSE) was recorded from the apical four-chamber view with the M-mode cursor positioned at the free wall angle of the tricuspid valve (TV) annulus [7]. RV long axis amplitude of motion (i.e. TAPSE) was measured from end-systolic to end-diastolic points [7], and its peak systolic velocity (S’) was measured from myocardial tissue Doppler imaging (TDI). All recordings of TAPSE and S’ were obtained during held end-expiration. Care was taken to align M-mode or TDI beam along the direction of tricuspid annulus motion, with the minimum angle in between. TDI sample volume was positioned at 10 mm from the insertion site of the tricuspid leaflets or 10 mm away within RV lateral wall and adjusted to cover the longitudinal excursion of the tricuspid annulus both in systole and diastole [5]. RVSD was defined as the presence of either TAPSE <16 mm or S’ of RV lateral tricuspid annulus <10 cm/s [6]. For the purpose of this study, recovery of RV systolic function was defined as an improvement of reduced TAPSE to ≥16 mm or S’ to ≥10 cm/s, without falling to reduced levels again, during follow-up.

Pulmonary artery systolic pressure (PASP) was estimated using continuous wave Doppler of the maximum velocity of the tricuspid regurgitant jet (v), from which the retrograde pressure drop was calculated using the modified Bernoulli equation (4 V^2^), and adding to it the estimated right atrial pressure (RAP) [8]. RAP was estimated using the diameter and collapse of the inferior vena cava during spontaneous respiration, as previously described [9]. Pulmonary hypertension (PHT) was defined as mean pulmonary arterial pressure (mPAP) of ≥25 mmHg at rest [10]. mPAP was estimated from PASP using the Chemla formula as: mPAP = (0.61 × PASP) +2 (mmHg) [11].

### Statistical analysis

Continuous variables were explored for the presence of skewness, which was corrected with logarithmic (log_10_) transformation. Patients’ baseline characteristics were described using frequencies and mean, while time period between patients’ delivery and recruitment was described using the median and inter-quartile range (IQR). Chi-square, Fisher’s exact probability and Student *t*-tests were used to compare categorical and continuous variables as appropriate. Spearman correlation coefficient (ρ_s_) was used to assess the association between TAPSE and mPAP and serum creatinine, while logistic regression models were used to assess the associations between RVSD or mortality and variables of interest. Linear regression was also used to assess the relationship between TAPSE and mPAP. Estimates for regression analyses were expressed as Odds Ratios (OR) and 95 % Confidence Intervals (CI). The regression results were tested with Hosmer and Lemeshow’s goodness of fit test, and a *p*-value ≥0.05 implied that the model’s estimates fit the data at an acceptable level. The statistical analysis was carried out using SPSS version 16.0 software. Two-sided *p*-value <0.05 was considered as minimum level of statistical significance.

## Results

**Fig. 1:**
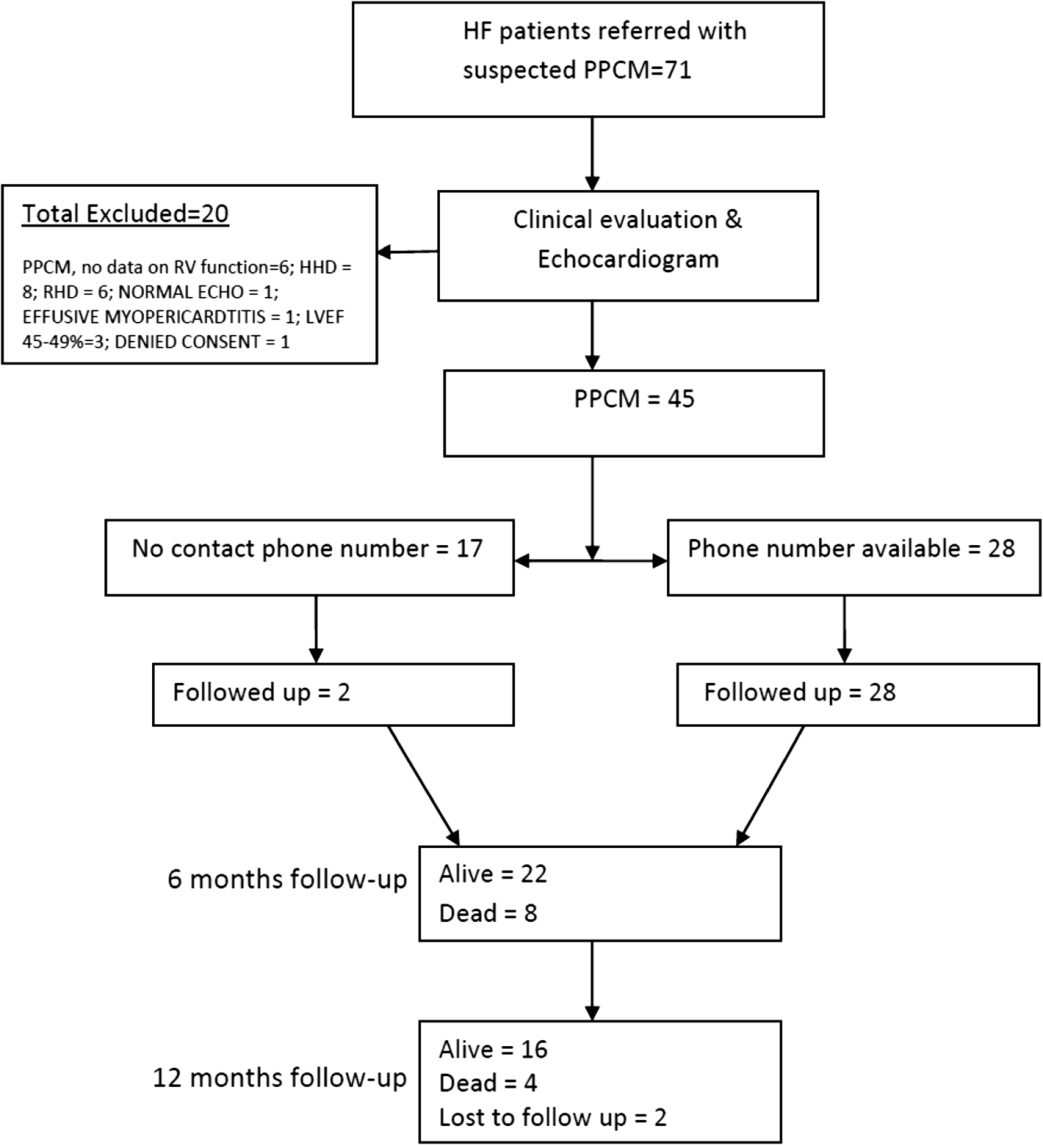
Flow chart of recruitment and follow-up of patients

The study flow is shown in Fig. 1. A total of 71 patients suspected to have PPCM were referred to the PI for possible inclusion, but only 51 (71.8 %) satisfied the diagnostic criteria, and all of them developed the disease postpartum. Of the 51 PPCM patients, 45 (88.2 %) had complete data for RVSD assessment at baseline, and of these 22 (48.9 %) were reviewed at 6 months, 8 (17.8 %) had died, while the remaining 15 (33.3 %) were lost to follow-up. At 1 year, 16/45 (35.6 %) patients were alive, 12 (26.7 %) had died and 17 (37.8 %) were lost to follow-up. The median time from delivery to recruitment for the patients was 6.0 (IQR: 3–15) weeks, and they were recruited in July to December 2013.

### Baseline clinical characteristics of patients with and without RVSD (Table 1)

**Table 1:** Baseline clinical characteristics of patients with and without RVSD

Variables	Patients with RVSD *N* = 32 (71.1 %)	Patients without RVSD *N* = 13 (28.9 %)	*p*-value
Age (years)	26.1 ± 7.5	27.7 ± 5.4	0.500
NYHA class:			0.809
II	13 (40.6 %)	6 (46.2 %)	
III	12 (37.5 %)	5 (38.5 %)	
IV	7 (21.9 %)	1 (7.7 %)	
Parity ≥2	25 (78.9 %)	10 (76.9 %)	0.758
Breastfeeding	30 (93.8 %)	13 (100 %)	–
BMI (Kg/m^2^)	21.4 ± 4.6	21.9 ± 3.9	0.704
Systolic BP (mmHg)	116 ± 23	128 ± 26	0.172
Diastolic BP (mmHg)	83 ± 18	94 ± 16	0.051
Heart rate/min	111 ± 16	104 ± 21	0.302
Pedal oedema	26 (81.3 %)	8 (61.5 %)	0.312
Hepatomegaly	19 (59.4 %)	6 (46.2 %)	0.515
Pregnancy associated hypertension	13 (40.6 %)	9 (69.2 %)	0.158
Haemoglobin (g/dL)	12.2 ± 1.8	12.9 ± 1.3	0.202
Log_10_ Creatinine	2.0 ± 0.2	1.9 ± 0.1	0.020*
Sodium (mmol/L)	136.3 ± 6.3	135.5 ± 5.2	0.655
Treatment:			
ACEI/ARB	15 (46.9 %)	7 (53.9 %)	0.749
Frusemide	32 (100 %)	13 (100 %)	–
Spironolactone	31 (96.9 %)	11 (84.6 %)	0.196
Digoxin	28 (87.57 %)	12 (92.3 %)	>0.999
Beta blockers	2 (6.3 %)	1 (7.7 %)	>0.999
Warfarin	1 (3.1 %)	1 (7.7 %)	0.499
α-Methyl Dopa	4 (12.5 %)	2 (15.4 %)	>0.999

Key: *NYHA* New York Heart Association functional classification, *BMI* body mass index, *ACEI* angiotensin concerting enzyme inhibitors, *ARB* angiotensin receptor blockers. Results are presented as means ± standard deviations, or as numbers with percentages in parentheses. * *p*-vlaue is statistically significant

Of the 45 recruited patients, 32 (71.1 %) had RVSD while the remaining 13 (28.9 %) had normal RV systolic function. When these 2 groups were compared, the differences were not statistically significant except for higher serum creatinine among those with RVSD (*p* = 0.02), who also tended to have lower diastolic blood pressure (DBP) (*p* = 0.05).

### RV remodelling

Baseline echocardiographic variables were compared between patients with and without RVSD in Table 2. Those with RVSD had significantly lower mean TAPSE and S’ (*p* < 0.001), but RV basal diameter, RAA, RAL, mPAP, and prevalence of PHT was not significantly different (*p* > 0.05) between the groups. Mean PAP was 29.3 ± 12.1 mmHg among all subjects respectively, out of whom a total of 30 (66.7 %) had PHT.

**Table 2:** Baseline echocardiographic characteristics of patients with and without RVSD

Variables	Patients with RVSD (*N* = 32)	Patients without RVSD (*N* = 13)	*p*-value
Right atrial end-systolic area, cm^2^	19.7 ± 7.6	15.8 ± 8.1	0.149
Right atrial length, mm	42.9 ± 10.2	42.5 ± 11.1	0.916
Right ventricular basal diameter, mm	49.6 ± 9.3	47.7 ± 10.7	0.583
TAPSE, mm	13.1 ± 2.6	20.3 ± 2.2	<0.001*
Right ventricular S’, cm/s	10.6 ± 3.2	15.3 ± 3.9	<0.001*
RIMP	1.09 ± 0.60	1.76 ± 1.30	0.032*
RV wall thickness, mm	3.6 ± 1.2	3.5 ± 0.8	0.952
Tricuspid Valve E:A ratio	1.21 ± 0.44	0.90 ± 0.35	0.019*
Tricuspid Valve E/e’	5.08 ± 2.73	3.46 ± 1.45	0.023*
Left atrial diameter, mm	43.2 ± 6.4	41.9 ± 6.2	0.548
LV end-diastolic diameter, mm	62.0 ± 7.5	62.4 ± 11.7	0.910
LV ejection fraction, %	32.4 ± 9.3	36.6 ± 7.7	0.127
LV stroke volume index, L/m^2^	39.9 ± 12.3	51.4 ± 17.9	0.017
Anti-log_10_ mitral valve E:A	1.50 ± 0.62	1.31 ± 0.25	0.154
Mitral valve E/e’	15.96 ± 7.13	15.61 ± 7.06	0.882
PA acceleration time, ms	61.8 ± 22.4	78.9 ± 28.0	0.037*
PA systolic pressure, mmHg	47.4 ± 19.4	39.2 ± 21.5	0.246
Mean PAP, mmHg	30.6 ± 11.6	25.9 ± 13.1	0.273
Pulmonary hypertension	23 (71.9 %)	7 (53.9 %)	0.304

Key: *TAPSE* Tricuspid annular plane systolic excursion, *LV* left ventricle, *E*:*A* transvalvular filling velocities ratio, *E*/*e*’ ratio of early filling to averaged early diastolic tissue Doppler velocities of mitral or tricuspid valves, *PAP* pulmonary artery pressure, *RIMP* right ventricular (RV) index of myocardial performance. Results are presented as means ± standard deviations, or as numbers with percentages in parentheses. *p-vlaue is statistically significant

**Fig. 2:**
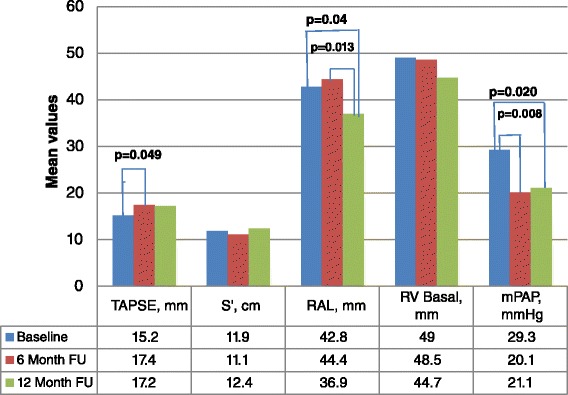
Pattern of RV remodelling among patients. Legend: *TAPSE* tricuspid annular plane systolic excursion, *RAL* right atrial length, *RV Basal* right ventricular basal diameter, *mPAP* mean pulmonary artery pressure, *FU* follow-up. Mean values of variables were computed and compared using Student *t*- test

The pattern of RV reverse remodelling is presented in Fig. 2, which shows increased TAPSE (*p* = 0.049) at 6 months, but RAL (*p* = 0.04) reduced at 12 months and mPAP fell at both 6 (*p* = 0.008) and 12 months (*p* = 0.020) follow up. The prevalence of RVSD reduced from 71.1 % at baseline to 36.4 % (8/22 patients) at 6 months (*p* = 0.007) and to 18.8 % (3/16 patients) at 12 months (*p* = 0.001 vs baseline and *p* = 0.41 vs 6 month). In addition, the prevalence of PHT fell to 36.4 % (*p* = 0.019) at 6 months and 31.3 % (*p* = 0.03) at 12 months. RV systolic function recovery occurred in a total of 8 patients (8/45; 17.8 %), of whom 6 (75.0 %) recovered in 6 months. Improvement in TAPSE alone was observed in 2 patients; in S’ alone in another 2 patients, while both TAPSE and S’ improved in 4 other patients.

**Fig. 3:**
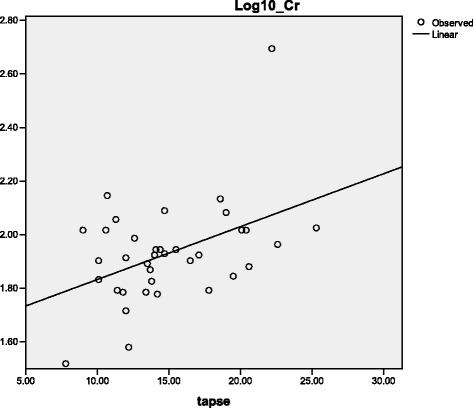
Relationship between TAPSE and serum creatinine among PPCM patients. Legend: Linear regression model showing that TAPSE accounted for 19.2 % (R^2^ = 19.2 %; *p* = 0.008) of the variability of serum creatinine

Further analysis showed that although baseline TAPSE was significantly associated with mPAP at 6 months follow-up (ρ_s_ = −0.531; *p* = 0.023), it did not predict its variability (R^2^ = 0.217; *p* = 0.051. Baseline TAPSE correlated with log_10_ creatinine (ρ_s_ = +0.332; *p* = 0.048), and accounted for 19.2 % (*p* = 0.008) of the variability of serum creatinine (Fig. 3). In addition, RVSD significantly increased the odds for log_10_ creatinine >1.95 (equivalent to serum creatinine 89.1 μmol/l) by 5.8 fold (OR = 5.83; CI = 1.263–26.944; *p* = 0.024).

When the baseline characteristics of subjects followed up were compared with those who were lost, differences between the groups were not statistically significant.

### RVSD and mortality

Of the 30 patients followed-up, 2 (6.7 %) were lost to follow-up and 12 died (40.0 %), of whom 8 (66.7 %) did so within the first 6 months. The deceased had a median survival time of 19.5 weeks. Of the 12 deceased patients, 10 (83.3 %) had RVSD while the remaining 2 (16.7 %) had normal RV systolic function (*p* = 0.47). Variables assessed in Tables 1 and 2 were compared between the deceased (12 subjects) and the survivors (16 subjects) at 1 year follow up, and the only significant difference between the groups was a lower serum haemoglobin level in the former (12.1 ± 1.3 g/dl) as compared to the latter (13.5 ± 1.4 g/dl) (*p* = 0.012). Step wise univariate regression analyses were then carried out in which the serum haemoglobin and the other variables in the Tables were assessed for possible association with 1 year mortality. However, the one year mortality wasn’t predicted by any variable in the univariate regression models, including RVSD (*p* = 0.284), serum creatinine (*p* = 0.441) and haemoglobin (*p* = 0.053) (Hosmer & Lemeshow test *Χ*^2^ = 9.69; *p* = 0.288).

## Discussion

The present longitudinal study assessed RVSD and RV remodelling and its response to treatment and potential recovery in a group of PPCM patients from Kano, Nigeria. The prevalence of RVSD, in the form of reduced TAPSE and RV free wall S’ velocity was evident in 71.1 % of the patients at baseline, and fell to 36.4 % at 6 months and to 18.8 % at 12 months follow-up. Likewise, PHT was found in 66.7 % of patients at baseline, and persisted in 36.4 % at 6 months, and to 31.3 % at 12 months follow up. RV systolic function recovery occurred in a total of 8 patients (8/45; 17.8 %), of whom 6 (75.0 %) recovered in 6 months. Forty percent of the followed-up patients died within 1 year; two-thirds of them within the first 6 months after diagnosis.

RVSD, its recovery and potential relationship with mortality are not well studied in PPCM. Based on reduced TAPSE, we have previously reported a prevalence of RVSD of 54.6 % in PPCM patients [3]. Adding RV reduced myocardial velocity (S’) raised the prevalence of patients with RVSD in this study to 71.1 %, suggesting a more accurate means for identifying such patients. The second important observation in the current study is the significant recovery of RVSD along with its pressure afterload in the form of PHT. Indeed, 6 months from the time of presentation, the prevalence of RVSD, PHT fell by more than 50 % despite poor adherence to heart failure conventional medications. Thus, the observed RV reverse remodelling seems to be related to the recovery of the pulmonary circulation status rather than to the effect of medications as has been previously observed in the LV [12]. This claim is supported by the modest relationship we found between TAPSE and mPAP at 6 months follow-up. Our finding might be explained by the significant RV function recovery in more than 50 % of patients at 6 months, thus suggesting a different pathophysiologic mechanism to right ventricular - pulmonary circulation coupling in PPCM compared with DCM, which has a more chronic course of disturbances of ventricular function [13]. In addition, TAPSE was linearly related to serum creatinine, confirming the adverse relationship between RVSD and renal impairment [14, 15]. Whether this reflects the aggressive nature of cardiac decompensation in PPCM, since all the patients developed LV systolic dysfunction with LVEF <45 % within a few postpartum days, remains to be verified in a larger group of patients.

Thus, our findings show that the first 6 months of PPCM diagnosis seem to be crucial for RV function recovery as well as survival. These findings are supported by Whitehead et al. who reported 87 % of PPCM deaths occurring within the first 6 months of diagnosis, and Elkayam who observed that LV function normalises in approximately 50 % of women with PPCM within the same period [12, 16]. These findings urge intensive medical treatment for such young patients within the first few days and weeks of their presentation. Finally, our mortality rate of 40 % in the present study seems high compared to 13 % over 6 months in South Africa [17]. This could be partly explained by the high maternal mortality in Nigeria in general, which was as high as 496.4 per 100,000 live births in 2013 [18].

### Clinical implications

In the absence of magnetic resonance imaging, which is known for its superior accuracy in assessing RV structure and function, bedside echocardiography has an acceptable accuracy for diagnosing and monitoring RV function in PPCM patients, and it is widely available and affordable, particularly in countries where PPCM is relatively more prevalent [19]. With the high mortality recorded in the present study, PPCM patients should receive intensive care in the acute phase, with thorough assessment and follow up of LV as well as RV function in an attempt to identify the vulnerable ones who need closer follow up.

### Study limitations

Although important, B-type natriuretic peptide (BNP) or N-terminal pro-BNP and other biomarkers could not be assessed because the tests were not readily available at the study centres. Secondly, it is important to point out that 15 of the 45 patients could not be followed-up because they were not contactable in spite of adequate counselling. Their inability to come for follow-up isn’t unrelated to the significant security challenges being faced by northern Nigeria including Kano, making movements and keeping to appointments extremely difficult. Thirdly, cardiac magnetic resonance imaging could have shed more light on the nature of the myocardial pathology in PPCM, but this facility was not available at the study centres [19].

## Conclusion

The present study assessed RVSD and pulmonary circulation disturbances in a group of PPCM patients in Kano, Nigeria. These were prevalent in over two-thirds of the patients at presentation and improved by more than 50 % at 6 months despite the lack of adherence to medical therapy. However, the studied cohort suffered a relatively high mortality which was not predicted by RVSD or other variables, in keeping with the overall high maternal mortality in Nigeria. These findings urge intensive care for such young patients during the acute phase of the disease to support cardiac function recovery and reduce mortality.

## Abbreviations

PPCM: peripartum cardiomyopathy
HF: heart failure
RV: right ventricle
TAPSE: tricuspid annular plane systolic excursion
RVSD: right ventricular systolic dysfunction
LV: left ventricle
MMSH: Murtala Mohammed specialist hospital
AKTH: Aminu Kano teaching hospital
LVEF: LV ejection fraction
PI: principal investigator
ECG: electrocardiogram
RAA: right atrial area
RVb: RV basal dimension
RAL: RA length
TV: tricuspid valve
TDI: tissue Doppler imaging
PASP: pulmonary artery systolic pressure
RAP: RA pressure
PHT: pulmonary hypertension
mPAP: mean pulmonary artery pressure
RIMP: right ventricular index of myocardial performance
ρ_s_: Spearman correlation coefficient
OR: odds ratio
CI: confidence interval
DBP: diastolic blood pressure
BNP: B-type natriuretic peptide
